# Characterizing Blood-Stage Antimalarial Drug MIC Values *In Vivo* Using Reinfection Patterns

**DOI:** 10.1128/AAC.02476-17

**Published:** 2018-06-26

**Authors:** James Watson, Cindy S. Chu, Joel Tarning, Nicholas J. White

**Affiliations:** a Mahidol-Oxford Tropical Medicine Research Unit, Faculty of Tropical Medicine, Mahidol University, Mae Sot, Thailand; b Centre for Tropical Medicine and Global Health, Nuffield Department of Medicine, University of Oxford, Oxford, United Kingdom; c Shoklo Malaria Research Unit, Mahidol—Oxford Tropical Medicine Research Unit, Faculty of Tropical Medicine, Mahidol University, Mae Sot, Thailand

**Keywords:** MIC, antimalarial agents, chloroquine, pharmacodynamics, pharmacokinetics

## Abstract

The MIC is an essential quantitative measure of the asexual blood-stage effect of an antimalarial drug. In areas of high malaria transmission, and thus frequent individual infection, patients who are treated with slowly eliminated antimalarials become reinfected as drug concentrations decline. In the frequent relapse forms of Plasmodium vivax and in Plasmodium ovale malaria, recurrent infection occurs from relapses which begin to emerge from the liver approximately 2 weeks after the primary illness. An important determinant of the interval from starting treatment of a symptomatic infection to the patency of these recurrent infections is the *in vivo* concentration-response relationship and thus the *in vivo* MIC. Using mechanistic knowledge of parasite asexual replication and the pharmacokinetic and pharmacodynamic properties of the antimalarial drugs, a generative statistical model was derived which relates the concentration-response relationship to time of reinfection patency. This model was used to estimate the *in vivo* MIC of chloroquine in the treatment of Plasmodium vivax malaria.

## INTRODUCTION

Careful characterization of the pharmacokinetic (PK) and pharmacodynamic (PD) properties of antimalarial drugs is necessary for the optimal design of malaria treatment regimens. Many antimalarial drugs have unusual pharmacokinetic properties, with very large apparent distribution volumes and long terminal elimination phases. The resultant protracted residual drug levels allow short-course treatments to be given (typically 3 days); they also provide a declining posttreatment prophylactic effect and thereby a disease-free interval during which malaria infections cannot become patent, and this affects the temporal pattern with which reinfections emerge (1, 2).

The MIC is an important quantitative measure of antimalarial drug effect in an individual infection (3). In the context of antimalarial chemotherapy, the MIC is defined as the theoretical average concentration of drug in whole blood or plasma which is associated with an observed parasite reduction ratio (PRR) of 1 per cycle. This implies that the rate of parasite killing due to the combination of drug and host immunity is equal to the rate of parasite multiplication and therefore reflects both parasite drug susceptibility and host defense. This interval between parasitemia reduction and increase is more evident when slowly eliminated antimalarial drugs are used. It should be noted that the term MIC has a different meaning in assessing antibacterial drugs. The antimalarial MIC is a therapeutic target which must be exceeded for a sufficient duration to ensure the elimination of the blood-stage malaria infection (4). Prospective estimation of the MIC *in vivo* in an individual infection is difficult, as it requires administration of inefficacious doses and serial measurement of submicroscopic parasite densities (5).

The method described here demonstrates how population MIC values can be estimated from observational data in higher transmission settings for falciparum malaria and frequent relapse settings for vivax malaria, where the follow-up period is long enough to detect the first recurrent infections.

## RESULTS

### MIC and the temporal patterns of recurrent infections.

While circulating drug concentrations are above the MIC (ρ_MIC_), all infections emerging from the liver following hepatic schizogony will be partially or totally suppressed; i.e., the asexual cycle (48-h) parasite reduction ratio is greater than 1, so parasite numbers decline. New infections usually emerge from the liver at a biomass of ≈10^4^ to 10^5^ parasites (the progeny of between 1 and 10 successful sporozoites) (6–8). As drug concentrations decline to ρ_MIC_ after drug administration, these emerging infections are suppressed by progressively smaller amounts until the MIC is reached, at which time point the parasite populations start to increase until, if drug levels decline sufficiently, their growth is an uninhibited first-order process with a multiplication rate which in nonimmune subjects usually approximates 8- to 10-fold per cycle (6, 9–11).

In a population of individuals in whom times to recurrent infection are measured by the same method (typically microscopy), this results in a “bunching up” or temporal clustering of initial recurrent infections reaching patency which are either newly acquired reinfections, relapses (in vivax malaria from dormant hypnozoites), or recrudescences (drug failures) (12, 13). If the antimalarial drug does not affect preerythrocytic development (the majority of antimalarials in current use do not have preerythrocytic activity), then the peak incidence of these recurrent infections will be at some time greater than the *t*_MIC_ plus 8 days (representing ≥4 asexual cycles). The precise timing depends on the terminal elimination half-life of the antimalarial drug and the slope of the concentration-response curve. If the drugs used do have liver-stage activity, then the bunching of the initial recurrent infections is delayed accordingly.

**FIG 1:**
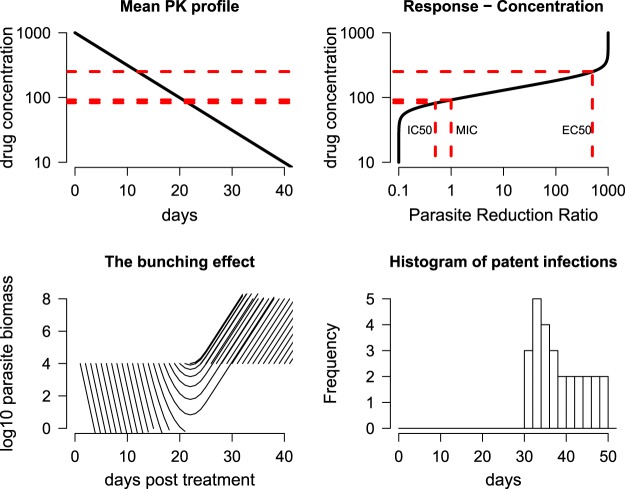
The theoretical dynamics of temporal clustering or bunching of new infections for a hypothetical slowly eliminated drug. (Top left) Mean pharmacokinetic profile of the drug, a first-order elimination with a half-life of 6 days. The IC_50_ (parasite reduction ratio of 1/5), MIC (parasite reduction ratio of 1), and EC_50_ (parasite reduction ratio of 500) are shown by dashed horizontal red lines. (Top right) Response-concentration function of the drug on a log_10_ scale. (Bottom left) time trajectories of total parasite biomass for daily new infections emerging from liver at a biomass of 10^4^ parasites where 10^8^ is the total parasite biomass in an adult associated with the onset of illness (pyrogenic threshold) and is also the lowest number of parasites readily detectable by microscopy. (Bottom right) Histogram of patent infections over time.

A hypothetical example illustrates this bunching phenomenon for falciparum malaria. Suppose a drug has a terminal elimination half-life of 6 days, starting concentrations (day 0) of 1,000 μg/liter, a maximum PRR of 10^3^, and a half-maximal effect (EC_50_) of 250 μg/liter, with a slope coefficient of the concentration-response curve of 7. The top panels in Fig. 1 show the pharmacokinetic-pharmacodynamic (PK-PD) model under these assumptions. The bottom left portion of Fig. 1 shows the fate of new infections acquired once daily (i.e., very high transmission intensity). This model shows why recurrent infections temporally bunch up together after antimalarial treatment (in this example, between days 30 and 38 [Fig. 1, bottom right]). The height and width of this peak reinfection time depend in theory on the slope of the concentration-response curve.

### Chloroquine for the treatment of Plasmodium vivax malaria on the Thailand-Myanmar border.

**FIG 2:**
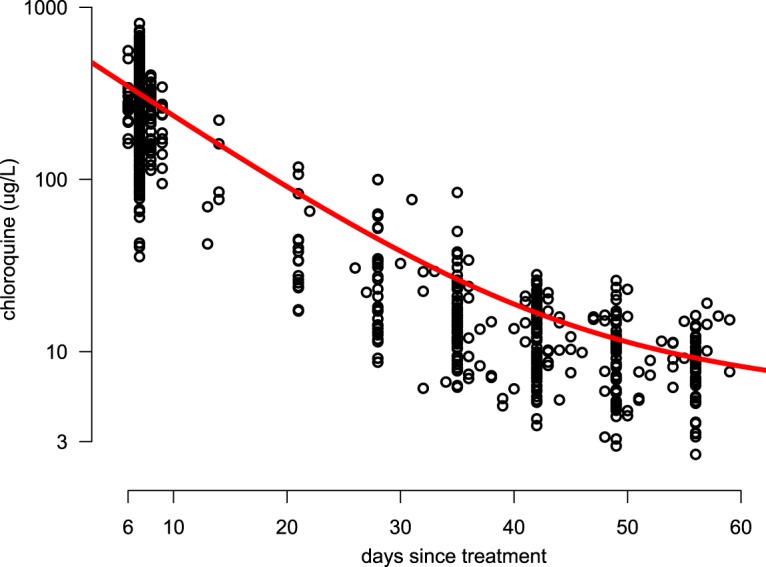
All pharmacokinetic samples up to day 60 from symptomatic patients with P. vivax malaria treated with chloroquine on the Thailand-Myanmar border. The whole-blood chloroquine concentration is plotted on a log_10_ scale. Outliers have been removed (see Materials and Methods).

A randomized controlled trial comparing therapeutic options for the treatment of vivax malaria was conducted by the Shoklo Malaria Research Unit on the northwest Thailand-Myanmar border (14). In the course of this study, a total of 418 intervals (in days) until a recurrent vivax malaria episode within 60 days from the previous episode were observed in 137 patients treated with standard doses of chloroquine (25 mg base/kg of body weight divided over 3 days). A mixed-effects biexponential first-order decay model described the population pharmacokinetics of chloroquine satisfactorily in all chloroquine-treated patients (*n* = 1,440 samples) (Fig. 2). The model estimated that approximately 97% of the drug is cleared rapidly (half-life at α1 phase [*t*_1/2_,α_1_], 6.7 days; population 95% interval, 5.3 to 9.3 days), and 3% of the drug is cleared slowly (*t*_1/2_,α_2_, 68 days; population 95% interval, 44 to 144 days).

**FIG 3:**
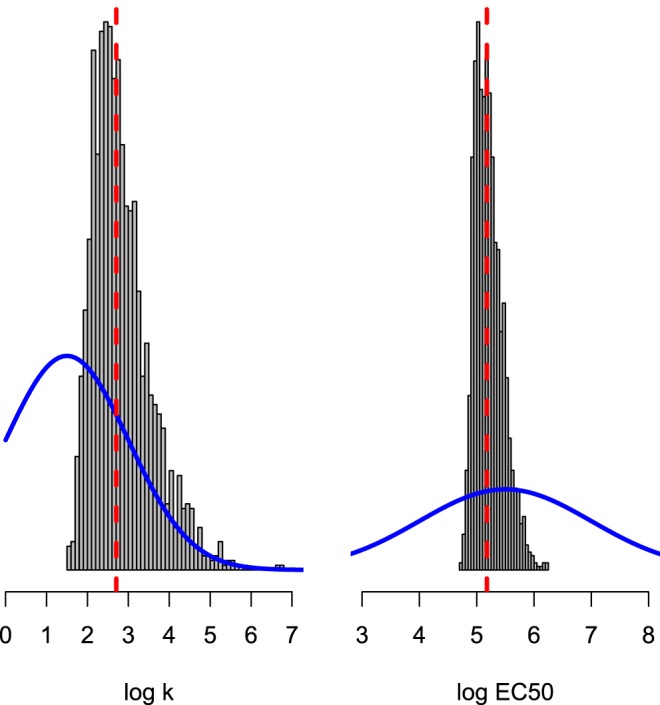
Prior and posterior distributions from ABC algorithm of concentration-response (PD) of chloroquine in vivax malaria patients from the Thai-Myanmar border. The algorithm estimates two pharmacodynamic parameters: the natural logarithm of the slope of the concentration-response (left) and the natural logarithm of the EC_50_ (right). The vertical dashed red line shows the median posterior value. The histogram shows the estimate of the posterior distribution and the thick blue line shows the prior distribution.

**FIG 4:**
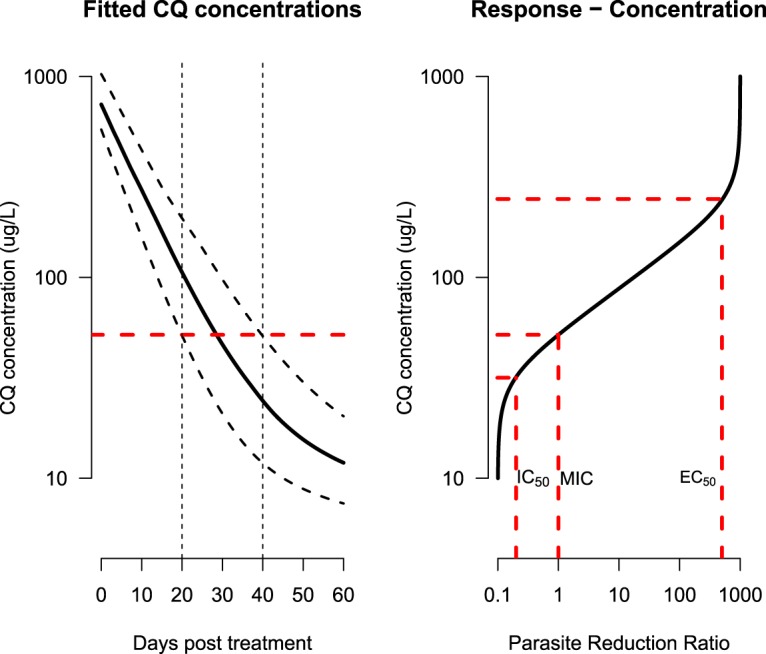
Estimated pharmacokinetics and pharmacodynamics of chloroquine (CQ) in the treatment of Plasmodium vivax in symptomatic patients from the Thailand-Myanmar border. (Left) fitted population pharmacokinetics showing median and 90% coverage intervals over time. The horizontal red dashed line shows the estimated whole-blood MIC of chloroquine in this series, with the vertical dashed lines showing the time window at which the whole blood drug concentrations of 90% of patients go below the MIC (14 to 29 days posttreatment). (Right) concentration-response curve for estimated parameter values. The locations of the IC_50_, MIC, and EC_50_ are shown by the dashed red lines.

In the context of this trial, the population whole-blood chloroquine MIC was estimated at approximately 67 μg/liter (95% posterior credible interval, 54 to 85 μg/liter). This corresponds to a concentration providing 50% of the maximum reduction of between 90 to 250 μg/liter and a slope coefficient of 5 to 30 (Fig. 3, slope coefficient shown on the log scale). Our method estimates that the average patient has residual drug concentrations above the MIC until approximately 20 days after starting treatment, with concentrations in 90% of patients going below the MIC between days 14 and 29 (Fig. 4, left). As a result, recurrent chloroquine-sensitive P. vivax does not usually become patent within 4 weeks of treatment. This is a week earlier than reported in the first studies of chloroquine for the treatment of P. vivax (15).

### Predicted interval-to-relapse phenotype of chloroquine-resistant P. vivax.

**FIG 5:**
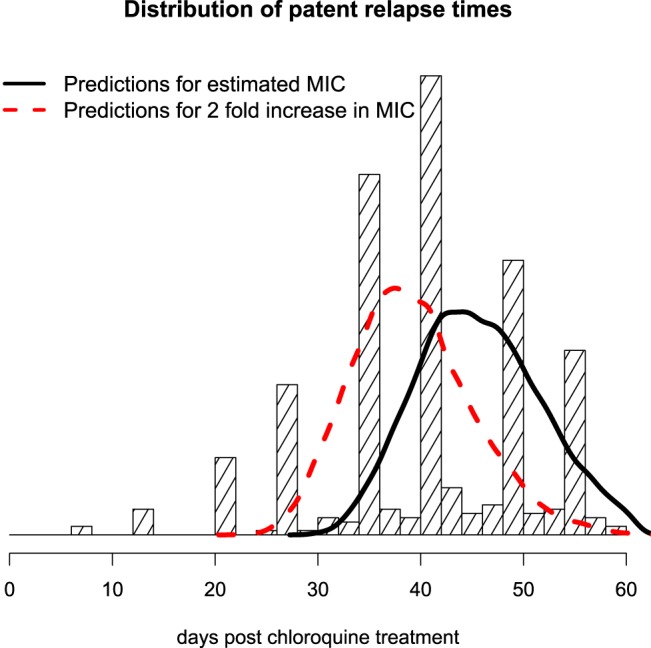
Predicting the shortening in the interval to patent relapse associated with reduced chloroquine susceptibility of P. vivax on the Thailand-Myanmar border. The observed distribution of times until detected relapse is shown by the histogram (note the effect of weekly active detection). The model fit of the distribution of relapse times is shown by the thick black line under current pharmacodynamic parameters. The model predicted distribution of relapse intervals with a doubling of the current estimated MIC parameter is shown by the dashed red line. This corresponds to median shift of 6 days.

If we make the assumption that chloroquine resistance can be quantified uniquely by a fold change in the MIC value (with no change to the slope parameter), then it is possible to predict how increasing resistance will affect the distribution of relapse intervals following chloroquine treatment. Our model predicts that a doubling of the MIC would result in the median time to relapse occurring approximately 6 days earlier (Fig. 5). In a more general setting, a doubling of the MIC value for slowly eliminated antimalarial drugs (with a monoexponential decline in concentrations over the relevant concentration range) should result in a shortening of the median time to relapse equivalent to one half-life of the drug (2).

### Implications for trial design.

The MIC of an antimalarial drug is a contextual parameter which is an important measure of drug efficacy and quantifies appropriate dosing. During the process of drug development, phase 2 trials specifically designed to estimate the MIC are already recommended, and their utility has been demonstrated (3, 5). We show that it is also possible to estimate the MIC from observational trial data in which slowly eliminated antimalarial drugs are used. In this example, Plasmodium vivax relapse was studied, but the same approach can be applied to Plasmodium falciparum infections in high-transmission settings provided that enough patients are studied. However, the following design aspects are important for the accuracy of the statistical estimation procedure. (i) There should be sufficient blood sampling to estimate the drug elimination profile. At a minimum this should be on day 7 and on the day of recurrent malaria if residual drug concentrations are still detectable. This depends on the drug studied. (ii) Follow-up should be for long enough to capture recurrent infections. A reasonable aim would be to observe 90% of the recurrences due to bunching. This depends on (*a*) the transmission rate (P. falciparum) or relapse rate (P. vivax) and (*b*) prior estimates of the pharmacokinetic-pharmacodynamic relationship (e.g., the duration of the average posttreatment prophylactic period). (iii) Parasite clearance rates for the primary illness should be documented to estimate the maximum *in vivo* effect. (iv) Sample size estimations should be performed by simulation using *a priori* knowledge of the transmission rate or relapse rate, the terminal elimination half-life of the relevant antimalarial drug, and its pharmacodynamic parameters.

## DISCUSSION

The antimalarial MIC is an individual value for a particular infection in a particular host. Ideally, in every treated patient antimalarial drug concentrations should exceed the MIC until the last blood-stage parasite has been killed. In practice, background immunity may clear infections which are not eliminated completely by the drug. But in any population background, immunity will vary widely and there will be some patients (notably young children) with little or no immunity. The MIC therefore provides a target allowing evidence-based rational design of treatment regimens (3). Unfortunately, the MIC is hard to measure in natural treated infections, yet the estimates derived in this context of real use, compared with animal models or subpatent human challenge models, are likely to be more relevant both to the treatment of malaria and to the selection of resistance. As MICs reflect host defense as well as parasite susceptibility, many of the values derived in areas of higher transmission may be lower than those in areas of lower transmission, where there should be little or no immunity to augment drug activity.

This modeling exercise illustrates one approach to field measurement that could be used in areas of high malaria transmission (or, indeed, in areas of lower transmission if very large numbers of patients are studied). It exploits the phenomenon of bunching, whereby infections newly acquired, or in this case relapsing, during and after the period of antimalarial treatment for an incident infection (or in a mass treatment) cluster together in time as they become patent. The precision of individual MIC estimates depends on several factors, including the frequency of patient observations and sampling, the accuracy of the pharmacokinetic estimates, the terminal elimination rate constant, and the frequency of reinfection. The pharmacokinetic estimates for an individual can be derived from as little as a single blood level (usually at day 7), with population estimates of the terminal elimination half-life obtained through weekly serial measurements. As many treatment studies involve weekly or less frequent outpatient follow-up and recurrent infections may be asymptomatic, the time to reinfection can be overestimated. If transmission is high, then partial immunity will result in many infections being cleared before they reach patency, and at lower transmission settings there will be substantial interindividual variation in the times when reinfections are acquired. Rapid reinfection (relative to the drug's terminal elimination half-life) is necessary for the implementation of this methodology. For example, long-latency P. vivax would not be a suitable candidate.

Although this approach could be incorporated in preregistration drug development, it is more suited to large studies of antimalarial drugs which have already been deployed. Phase 3 antimalarial drug studies evaluating asexual blood-stage activity may not enroll a sufficient number of patients in an appropriate setting, e.g., children, in whom P. falciparum reinfection rates are high, or patients with high rates of P. vivax relapse, to provide accurate MIC estimates. Thus, the method may be more useful for postregistration dose adjustments (which have happened for most of the antimalarial drugs in current use) and for the interpretation of resistance. As the MIC reflects both host immunity and parasite susceptibility, it offers a method for assessing the former (by comparison between sites and ages) and calibrating the latter (by comparing *in vivo* and *in vitro* assessments from the same site).

In this study, the estimated *in vivo* whole-blood chloroquine MIC for Plasmodium vivax on the Thailand-Myanmar border was approximately 67 μg/liter (95% posterior credible interval, 54 to 85 μg/liter), which may be compared with a recent *in vitro* geometric mean *ex vivo* 50% inhibitory concentration (IC_50_) estimate of 17.3 μg/liter (95% confidence interval, 16.2 to 19.84 μg/liter). Given that whole-blood chloroquine concentrations are 8 to 10 times higher than in plasma samples, and that chloroquine is approximately 50% protein bound, these values are not very different (16, 17).

It is assumed that resistance is not a significant problem yet (although in the example studied, there is some evidence for emerging chloroquine resistance) and so the minority of newly acquired or relapsing infections which do become patent are no different in terms of antimalarial drug susceptibility from those which emerged earlier and were cleared spontaneously. These sources of variability are compounded by variation in the duration of preerythrocytic schizont maturation, variation in the number of successful sporozoites inoculated, and variation in parasite growth rates. Nevertheless, if there are sufficient numbers of observations to observe bunching, MIC estimates can be provided from field studies in which patient follow-up is frequent and sufficiently long to capture recurrences and antimalarial blood concentrations have been measured.

## MATERIALS AND METHODS

All statistical analysis and modeling was done with R software (version 3.4.1). Reproducible stand-alone R code and data used for the chloroquine modeling can be found at https://github.com/jwatowatson/bunching.

### Pharmacokinetic-pharmacodynamic model.

This section presents the theoretical framework which elucidates the relationship between the temporal pattern of recurrent infections and the MIC, or time to reach MIC. The MIC is the estimated antimalarial drug concentration at which the parasite multiplication rate/cycle is one. If the parasite multiplication rate is 10 per blood-stage asexual cycle, then this would correspond to 90% inhibition of multiplication. This approach to MIC estimation relates to slowly cleared antimalarial drugs (typical of artemisinin partner drugs in current combination treatments). The dynamics of the model show why the first recurrent infections to become patent after the administration of slowly eliminated antimalarial treatments cluster together temporarily and how this bunching effect is partially determined by the MIC.

Many antimalarial drugs have multiphasic concentration profiles during the distribution and elimination phases. However, the terminal elimination profile is usually a first-order process characterized by a terminal elimination rate constant which is inversely proportional to the terminal elimination half-life. This varies in a population of treated patients because of population variation in pharmacokinetics.

The pharmacokinetic-pharmacodynamic model defines the relationship between concentrations of the drug (as measured in whole blood or plasma) and the asexual parasite reduction ratio per 48-h cycle, i.e., the schizonticidal effect of the drug. Parameter estimation is done by discretizing into 24-h intervals. Because it is only the schizonticidal effect which is of interest, the setups of the PK-PD model are identical for both Plasmodium falciparum and P. vivax and are independent of the source of parasitemia, whether a reinfection, a recrudescence, or a relapse in the case of P. vivax.

### Pharmacodynamic model.

In this exercise, the pharmacodynamic outcome was the parasite reduction ratio over the course of one asexual blood-stage life cycle (48 h). This is a positive real number which takes values greater than 1 when the parasite population declines and values less than 1 when the parasite population grows. Framing the problem in this way allows for population dynamics to be characterized with a single continuous outcome. We assume that the antimalarial concentration-effect relationship is a sigmoid function of the log concentration of drug in the blood or plasma. This sigmoid function is parameterized by its linearized slope coefficient (which we designate *k*), the drug's maximal effect *L*_max_ (18, 19), the drug's minimal effect *L*_min_, which corresponds to maximum parasite growth rates in the presence of negligible drug concentrations, and one particular value of the concentration-response curve. In this exercise, we use the *in vivo* half-maximal effective concentration (EC_50_), which we designate ρ_1/2_. Other suitable choices would include the EC_50_ or the half-maximal inhibitory concentration (IC_50_). The choice of parameter should reflect best prior knowledge of the corresponding concentration-response curve. We argue that the EC_50_ or the EC_90_ will most often be points on the concentration-response with the most accurate *a priori* knowledge. These are all *in vivo* measures and have different values when assessed *in vitro* using different endpoints.

### Definition 1: MIC.

The MIC is defined as the theoretical concentration of drug at which the parasite reduction ratio is exactly 1 over the course of one life cycle.

### Definition 2: EC_50_.

The EC_50_ is defined as the theoretical concentration of drug at which the blood-stage parasite reduction ratio is *L*_max_/2 over the course of one life cycle. This is the concentration resulting in half the maximal reduction in parasite numbers per asexual cycle (e.g., approximately 500 for chloroquine).

### Definition 3: IC_50_.

The IC_50_ is defined as the theoretical concentration of drug at which the parasite reduction ratio is *L*_min_/2 over the course of one life cycle. This is the concentration causing half the maximal growth per asexual cycle (e.g., 5-fold growth per cycle if the maximum average growth rate was 10-fold/cycle).

The four parameters (θ_PD_ = {*L*_max_, *L*_min_, *k*, ρ_1/2_}) uniquely define the pharmacodynamic properties of the drug. A commonly used mathematical formula for this sigmoid relationship is given by the logistic function. For example, the 48-h parasite reduction ratio as a function of the logarithm of the drug concentration ρ can be written as

$$\text{PRR}(\rho )=L_{\min}+\frac{L_{\max}-L_{\min}}{1+e^{-k\;(\rho -\;\rho_{\frac{1}{2}})}}$$

We note that the slope of this sigmoid curve at the midpoint is given by *k*/4. Empirical data, both *in vivo* and *in vitro*, can usually give a robust characterization of the central aspect of this curve, i.e., around the ρ_1/2_ and concentrations at which the effect is close to *L*_max_ (maximal effect) (20). However, the relationship between *in vitro* and *in vivo* measures has not been well characterized, and direct measurement *in vivo* is very difficult (5). The MIC is determined by the parameters {*L*_max_, *L*_min_, *k*, ρ_1/2_}, but small errors in *k* will have a large impact on the estimation of ρ_MIC_. This can be observed by the relationship:

$$\rho_{\text{MIC}}=\rho_{1/2}-\frac{\log(L_{\max}-1)-\log(1-L_{\min})}{k}$$

where ρ_MIC_ is the logarithmic concentration of drug which gives a PRR of 1 and ρ_1/2_ is also on a logarithmic scale.

In the following we assume that *L*_min_ is known: this is the reciprocal of the normal parasite growth rate with negligible drug concentrations. In the estimation algorithm we set *L*_min_ = 0.1 (e.g., parasite growth rate of 10-fold per 48-h cycle). This value is independent of the drug studied.

We also assume that the *L*_max_ is known for chloroquine and is 10^3^.

### Statistical inference.

This section outlines the Bayesian statistical method we use for the estimation of the *in vivo* MIC using data on intervals to recurrent infection. A Bayesian approach for the estimation of the MIC is appropriate, as there will be informative prior information regarding the key parameters in the model. For example, *in vitro* dose-response data can provide plausible values for the slope of the dose-response curve, although the EC_50_ (ρ_1/2_) will in general not be the same.

This statistical model makes the following assumptions: (i) the drug lacks preerythrocytic activity and (ii) there are no recrudescences; i.e., the time to reinfection *T_i_* has to be greater than 8 days. (Preerythrocytic activity can also be incorporated if the pharmacodynamic activities for liver- and blood-stage antimalarial activities have similar properties. Recrudescences can be incorporated if genotyping is performed to distinguish recrudescence from reinfection.)

Let *T*_1, …,_ *T_n_* be the times of posttreatment malaria recurrences (vivax or falciparum) for *n* patients, for a given antimalarial. Let $\rho_{0:T_{i}}^{\left(i\right)}$ be logarithmic drug concentrations for patient *i* from treatment up to *T_i_*. In practice, these may either be simulated from a population PK model or extrapolated from patient measurements relevant to the terminal phase of the drug (for example, day 7 measurements). The imputation of the drug concentrations over time is considered a separate inference problem. Thus, the logarithmic concentrations $\rho_{0:T_{i}}^{\left(i\right)}$ are considered observed (i.e., data and not parameters of the model to be estimated). We assume that the times to recurrence are independently distributed given the population PD parameters θ_PD_ and the logarithmic drug concentrations $\rho_{0:T_{i}}^{\left(i\right)}$. Let *S_i_* be the (unknown) time of hepatic schizont rupture.

We define a generative model which computes the time of reinfection (patent reinfection, in days) *Y_i_* conditional on a value of $\left\{\theta_{\text{PD}},\;\rho_{0:T_{i}}^{\left(i\right)},\;S_{i}\right\}$. The value of *Y_i_* is computed by solving

(1) $$Y_{i}=S_{i}+2\times \arg\min_{T}\;\{{\text{biomass}}_{\text{pyrogenic}}\leq 10^{4}\times \overset{T}{\underset{c=1}{\Pi }}1/\text{PRR}(\rho_{s_{i+2c}}^{\left(i\right)}|\theta_{\text{PD}})\}$$

where biomass_pyrogenic_ is the threshold total number of blood-stage asexual parasites causing fever in an adult (the pyrogenic density), usually above 10^8^, 10^4^ is the number of parasites emerging from the liver for each inoculated sporozoite, and $\text{PRR}(\rho_{{\overset{\left(i\right)}{S}}_{i+2c}}|\theta_{\text{PD}})$ is the parasite reduction ratio for circulating drug concentrations $\rho_{{\overset{\left(i\right)}{S}}_{i+2c}}$, and where *c* indexes the number of parasite asexual cycles after hepatic schizont rupture. If *S_i_* occurs too early and the infection is cleared (i.e., the number of parasites goes below 1), then *Y_i_* is set to 0.

### ABC-type algorithm.

We use the following approximate Bayesian computation (ABC)-type algorithm (21) to estimate the posterior distribution of θ_PD_.

1. Repeat for *k* = 1,..,K samples.
  Sample $\theta_{\text{PD}}^{k}\;∼\;\Pi (\cdot )$.
  For subjects *i* ∈ 1..*N*:
    Sample latent variable $S_{i}\;∼\;F(\cdot )$.
    Simulate forward the fate of the infection (discretizing daily) as in equation 1 with PD parameters fixed as $\theta_{\text{PD}}^{k}$ and latent merozoite release time *S_i_* until infection is cleared or total number of parasites goes above pyrogenic threshold.
    Save time of reinfection $Y_{k}$ (zero if infection is cleared).
  Compute the *L*_1_ distance between observed and simulated data:
    $$L_{k}=\overset{N}{\underset{i=1}{\sum }}\left|Y_{k}^{i}-T_{i}\right|$$

1. Select closest samples.
  A. Select all samples $\theta_{\text{PD}}^{k}$ such that *L_k_* < ε.

In this algorithm, Π is the prior distribution over the parameters $\theta_{\text{PD}}^{k}$; *F*(·) is the prior distribution over merozoite release times: the latent variables $S_{i}^{k}$.

### Incorporating unknown drug concentration profiles.

In most antimalarial clinical trials, the drug concentration profiles $\rho_{0:T_{i}}^{\left(i\right)}$ for each patient will not be known with good precision. Many trials now take day 7 measurements of the drug concentrations of slowly eliminated drugs and metabolites; however, population variation in the terminal elimination half-life *t*_1/2_ affect the precision of the posterior estimates of ρ_MIC_. It is easy to augment the statistical model with a hierarchical prior over values of $t_{1/2}^{\left(i\right)}$, the *i*th patient individual elimination half-life. For example, the terminal elimination half-life of lumefantrine varies between 3 to 6 days, so one option would be a Gaussian prior with a mean of 4.5 days and standard deviation of 0.75 day (22). This augments the parameter space by adding *n* new parameters. To avoid overfitting, another solution is to compute a biased posterior distribution with imputed data $\rho_{0:T_{i}}^{\left(i\right)}$ using the population median/mean terminal half-life.

### Overview of randomized trial of chloroquine versus artesunate monotherapy.

In a randomized controlled trial between May 2010 and October 2012, patients older than 6 months and weighing more than 7 kg with microscopy confirmed uncomplicated P. vivax monoinfection were randomized to receive artesunate (2 mg/kg/day for 5 days), chloroquine (25 mg base/kg divided over 3 days: 10 mg/kg, 10 mg/kg, and 5 mg/kg), or chloroquine plus primaquine (0.5 mg base/kg/day for 14 days). All doses were supervised by the study staff. Informed consent was obtained from all patients. Ethical approval of the study was given by the Mahidol University Faculty of Tropical Medicine Ethics Committee (MUTM 2010-006) and the Oxford Tropical Research Ethics Committee (OXTREC 04-10). See clinicaltrials.gov trial number NCT01074905 (14).

Subjects were followed daily for supervised drug treatment. Follow-up continued weekly for 8 weeks and then every 4 weeks for a total of 1 year. Sampling for chloroquine levels (capillary whole blood) was done on day 6 (±1 day) and on the day of a recurrence. Recurrent episodes were detected actively at scheduled visits by microscopy (lower limit of detection is circa 20 parasites per μl). Patients were encouraged to come to the clinics between scheduled visits when unwell, so some recurrences would have been detected passively. When recurrence occurred with microscopy-confirmed P. vivax, the same study activities as on the day of enrollment were performed, with the addition in two of the study arms of a capillary sample for the measurement of blood chloroquine levels. The patient was retreated with the same study drug as in the original allocation. For vivax malaria, recurrent symptomatic or asymptomatic episodes could originate from failure of the drug to eliminate every last parasite (termed recrudescence), a reinfection from a new mosquito inoculation (termed reinfection), or activation of dormant parasites in the liver known as hypnozoites (termed relapse). In practice, most or all early recurrences are relapses.

For the purposes of this analysis, we used only the data from individuals randomized to the artesunate and chloroquine monotherapy arms. Times until relapse combined with estimated PK profiles for chloroquine treated patients are used to estimate the MIC of chloroquine. Times until relapse and total parasite biomass for artesunate monotherapy-treated patients were used to derive a prior distribution over liver schizont rupture times.

### Data cleaning and preparation. (i) Data used for MIC estimation.

The data used to estimate the whole-blood MIC of chloroquine consisted of 418 recurrences before day 60 occurring in 137 individuals (95 males and 42 females). The number of recurrences ranged from 1 to 10, with a median number of recurrences of 2. The median time to recurrent infection in this subset of patients treated with chloroquine was 42 days. Patient ages ranged from 1.5 to 55 years (median age was 18 years).

To estimate the chloroquine pharmacokinetic profiles of these patients, we pooled data from all chloroquine-treated patients, giving a total of 1,440 PK samples from 438 individuals. The median number of samples per individual was 1.

### (ii) Calculation of times since merozoite release.

Our PK-PD model of chloroquine and P. vivax parasites depends on the latent time of merozoite release into blood. The following details how total parasite biomass is estimated for each individual and the data from the artesunate monotherapy arm are used to obtain a prior distribution of times of merozoite release.

Total parasite biomass is estimated as follows: biomass = parasites per microliter × 10^6 ×^ BV, where BV is the patient's blood volume, estimated as 2.78 liters/m^2^ for males and 2.44 liters/m^2^ for females (23). Surface area for each patient was calculated using the Du Bois formula (body surface area [BSA] = weight^0.425^ × height^0.725^ × 71.84 × 10^−4^). The parasite count used is the one recorded on detection of each new episode.

Artesunate is a rapidly eliminated drug (active metabolite dihydroartemisinin half-life < 1 h) and thus has no posttreatment prophylactic effect. Therefore, in patients randomized to artesunate monotherapy, the time (since last treatment) of relapse is indicative of when liver merozoites were released into the blood. We assume that in the absence of drug (or at negligible concentrations) the parasite multiplication rate is 10-fold per 48-h cycle. We assume that each new bloodstream infection is initiated by 10^4^ liver merozoites. The number of days since merozoite release is then estimated as

$$2\times \log_{10}(\frac{\text{parasites}\;\text{per}\;\mathrm{\mu L}\times 10^{6}\times \text{BV}}{10^{4}})$$

This gives an estimated distribution of times of merozoite release. This distribution is then approximated by a two-parameter continuous Weibull distribution which is used as a prior distribution to generate latent merozoite release times in the generative statistical model. A larger initial inoculum results in a shorter interval; e.g., if the infection is initiated by 10^5^ merozoites, then the interval is 2 days shorter.

### (iii) Removal of outliers.

Outlier detection was done for samples taken around day 6 with very low concentrations (below 100 μg/liter). We also removed samples taken after day 60, as we consider recurrent infections only before day 60 to be informative for the MIC. Day 60 was chosen as a qualitative assessment based on the difference in the distribution to time to recurrence between artesunate-treated individuals (no postprophylactic effect) and chloroquine-treated individuals.

### Pharmacokinetic analysis.

A mixed-effects biexponential decay model was fitted to these PK samples (R software, function *nlme* from package *nlme*). The concentration of chloroquine at time *t* is designated ρ(*t*) and is modeled as

(2) $$\rho (t)=\beta (p\;e^{-\alpha_{1^{t}}}+(1-p)e^{-\alpha_{2^{t}}})$$

where β is the intercept term (peak concentration after dosing which is assumed to be time zero), *p* is the proportion defining the biexponential decay and α_1_ and α_2_ are the two decay rates. To avoid overfitting, *p* was assumed to be a fixed effect. The intercept β and the rates α_1_ and α_2_ were assumed to be random effects.

The following data and metadata on all subjects included in this analysis are available at https://github.com/jwatowatson/bunching along with the model code: the time to relapse (in days), the estimated total parasite biomass, and the individual parameter fits from equation 2, which gives the whole fitted PK profile over time.

### MIC estimation method.

We use the ABC algorithm outlined under “Statistical inference” above, with the following specifics.

- The latent merozoite release times for each reinfection time *T_i_* (*i* = 1..418) is generated from a truncated Weibull distribution (shape = 2.12, scale = 19.86) where the lower bound is zero and the upper bound is the time of recurrent infection minus 6 days. The parameters of the Weibull distribution were estimated from the artesunate-treated individuals (see above).
- The maximum PRR of chloroquine was fixed at 10^3^.
- The prior over θ_PD_ was set as a bivariate normal distribution over the natural logarithm of the slope and the IC_50_, with means 1.5 and 5.5, respectively. For both parameters the standard deviation was set to 1.5 (Fig. 5).
- Point estimates of the PK profile for each individual were taken from a mixed-effects model (as in “Data cleaning and preparation” above) and plugged into the ABC algorithm.
- Total parasite biomass corresponding to patency of infection was taken as the individual estimated parasite biomass on detection of episode (this is parasite count per microliter multiplied by total blood volume calculated from estimated body surface area).

We ran 10^5^ iterations of the algorithm. The posterior distributions of the PD parameters are shown in Fig. 3.
